# Correlation between the area of high-signal intensity on SPIO-enhanced MR imaging and the pathologic size of sentinel node metastases in breast cancer patients with positive sentinel nodes

**DOI:** 10.1186/1471-2342-13-32

**Published:** 2013-09-13

**Authors:** Kazuyoshi Motomura, Tetsuta Izumi, Souichirou Tateishi, Hiroshi Sumino, Atsushi Noguchi, Takashi Horinouchi, Katsuyuki Nakanishi

**Affiliations:** 1 Department of Surgery, Osaka Medical Center for Cancer and Cardiovascular Diseases, 1-3-3 Nakamichi, Higashinari-ku, Osaka 537-8511, Japan; 2 Department of Radiology, Osaka Medical Center for Cancer and Cardiovascular Diseases, Osaka, Japan

**Keywords:** Sentinel node, Breast cancer, Magnetic resonance imaging, Computed tomography, Superparamagnetic iron oxide, Nodal enhancement pattern, Lymph node metastasis

## Abstract

**Background:**

We previously demonstrated that superparamagnetic iron oxide (SPIO)-enhanced MR imaging is promising for the detection of metastases in sentinel nodes localized by CT-lymphography in patients with breast cancer. The purpose of this study was to determine the predictive criteria of the size of nodal metastases with SPIO-enhanced MR imaging in breast cancer, with histopathologic findings as reference standard.

**Methods:**

This study included 150 patients with breast cancer. The patterns of SPIO uptake for positive sentinel nodes were classified into three; uniform high-signal intensity, partial high-signal intensity involving ≥50% of the node, and partial high-signal intensity involving <50% of the node. Imaging results were correlated with histopathologic findings.

**Results:**

Thirty-three pathologically positive sentinel nodes from 30 patients were evaluated. High-signal intensity patterns that were uniform or involved ≥50% of the node were observed in 23 nodes that contained macro-metastases and no node that contained micro-metastases, while high-signal intensity patterns involving <50% of the node were observed in 2 nodes that contained macro-metastases and 8 nodes that contained micro-metastases. When the area of high-signal intensity was compared with the pathological size of the metastases, a pathologic >2 mm sentinel node metastases correlated with the area of high-signal intensity, however, a pathologic ≤2 mm sentinel node metastases did not.

**Conclusions:**

High-signal intensity patterns that are uniform or involve ≥50% of the node are features of nodes with macro-metastases. The area of high-signal intensity correlated with the pathological size of metastases for nodes with metastases >2 mm in this series.

## Background

Sentinel node biopsy is emerging as an alternative to axillary lymph node dissection for patients with breast cancer with clinically negative nodes [1-5]. It is associated with less morbidity, such as lymph edema and neuropathies, than axillary lymph node dissection, but with high accuracy in the prediction of axillary nodal status; however, it still involves a surgical procedure with associated morbidity. Overall, 2-7% of patients are reported to have lymphedema even after sentinel node biopsy [6-8].

Recently, intravenously administered ultrasmall superparamagnetic iron oxide (USPIO)-enhanced MR imaging has been reported to be promising for the diagnosis of lymph node metastases as a noninvasive method. Some researchers have already evaluated nodal staging in various tumors [9-11]. Harisinghani et al. demonstrated a sensitivity of 100% with a specificity of 95.7% for nodal staging using USPIO-enhanced MR imaging in 80 patients with prostate cancer [9]. Rockall et al. reported that the sensitivity for nodal staging using USPIO-enhanced MR imaging in 768 lymph nodes from 44 patients with endometrial or cervical cancer was 100%, significantly higher than the conventional method using size criteria, which has a sensitivity of 27% [10]. Stets et al. reported an accuracy of 87%, sensitivity of 81%, and specificity of 92% using USPIO-enhanced MR imaging for axillary lymph node metastases in 52 lymph nodes from 9 patients with breast cancer [11]. A recent meta-analysis demonstrated that USPIO-enhanced MR imaging is sensitive and specific, and superior to other modalities in the detection of nodal metastases for various malignancies [12].

We previously assessed MR imaging using interstitial injection of superparamagnetic iron oxide (SPIO) enhancement for the detection of metastases in sentinel nodes which were localized by CT-lymphography (CT-LG) in patients with breast cancer [13]. We demonstrated that SPIO-enhanced MR imaging accurately stages the axilla and may avoid even sentinel node biopsy in patients with breast cancer.

In the present study, we investigated the correlation between the area of high-signal intensity on SPIO-enhanced MR imaging and the pathologic size of sentinel node metastases in breast cancer patients with pathologically positive sentinel nodes for determining the predictive criteria of the size of nodal metastases.

## Patients and methods

### Patient selection

One hundred and fifty consecutive patients with clinical T1-2 breast cancers and clinically negative nodes who underwent sentinel node biopsy at Osaka Medical Center for Cancer and Cardiovascular Diseases between January 2008 and November 2009 were enrolled in this study. Patients with nonpalpable breast cancer, prior axillary surgery or who were pregnant were excluded. Patients with a contraindication to CT or MR imaging, or a known allergy to the contrast agents were also excluded. The institutional review board of Osaka Medical Center for Cancer and Cardiovascular Diseases approved the study, and written consent was obtained from all patients.

### Sentinel node localization using CT-LG

Interstitial CT-LG was performed using a multidetector row helical CT scanner (Light Speed VCT; GE Healthcare, Milwaukee, WI, USA). Contiguous 1.25-mm-thick CT images from the upper thorax to axillary regions were obtained once before administration of the contrast agent. CT scanning with a detector of 0.625 mm, 64 rows was operated at 120 kV, 300 to 400 Auto-mA, 35 cm field of view, 512 × 512 matrix, section spacing of 1 mm, and a table speed of 1.55 mm/0.5 sec.

Transaxial CT images were reconstructed with a 1.25-mm pitch and slice thickness of 0.3 mm. Each patient was placed in the supine position. Three small plastic bullets were placed as landmarks on the upper chest wall on the skin for a merged image of the CT-LG and axial MR image. First, their arms were placed in an elevated position. After local anesthesia with a subcutaneous injection of 2 ml of 2% procaine hydrochloride, 6 ml iopamidol (Iopamiron 370; Bayer Schering Pharma, Osaka, Japan) was injected intradermally into the skin overlying the breast tumor and into the subareolar skin. A CT scan was performed after massaging the iopamidol injection site for one minute. Second, a CT scan was performed in the adducted arm position. Finally, their arms were placed in an elevated position again, and a localizing marker, which is usually used for CT-guided lung nodule biopsy, was attached to the skin at the axilla to identify the sentinel node location over the skin [14]. The sentinel node location was identified on the CT image and was indicated precisely by the crossing point of the localizing marker and the CT plane lights. The site was marked on the skin surface using an oil pen.

**Figure 1:**
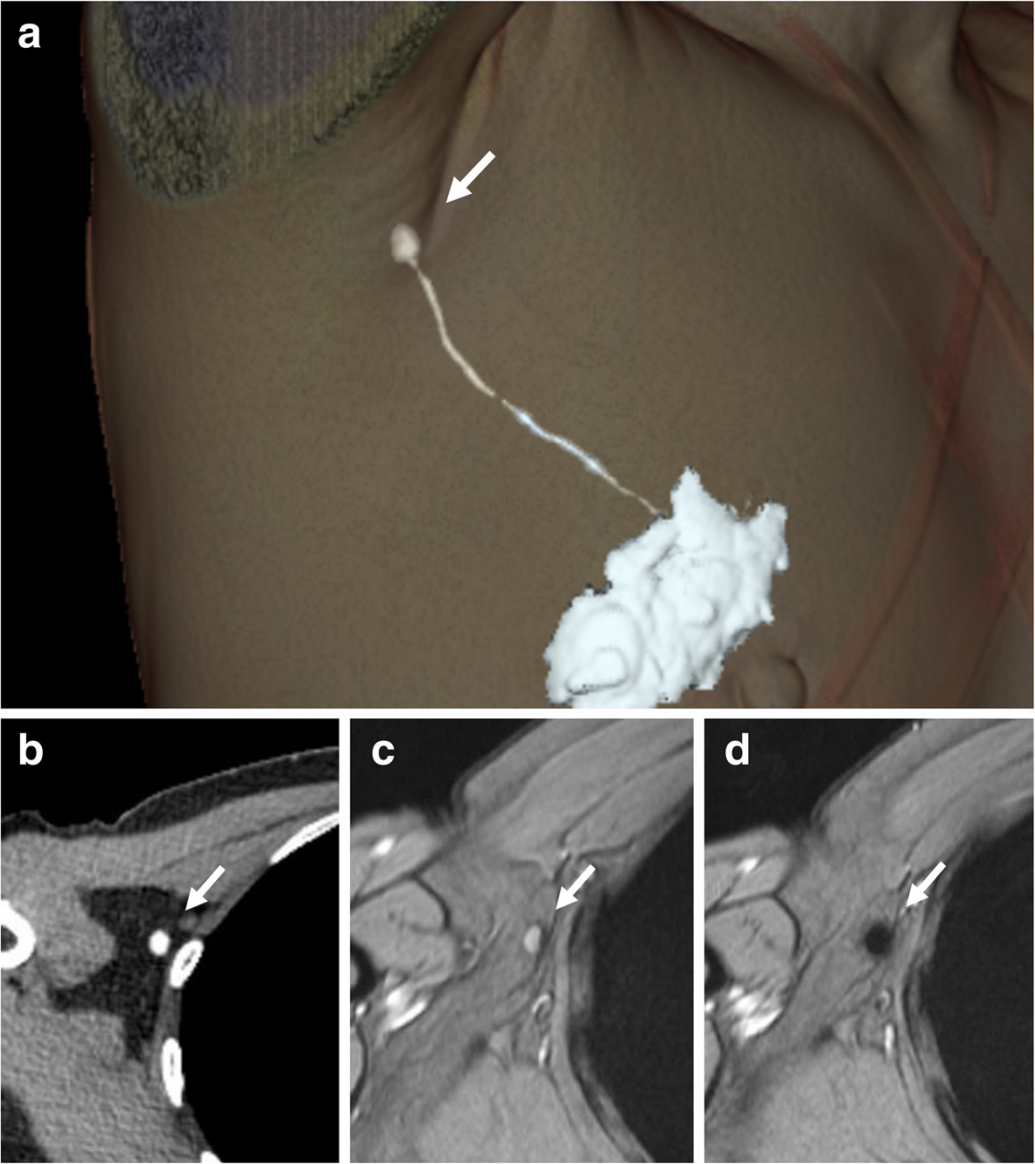
**Sentinel node localization using CT-lymphography and SPIO-enhanced MR imaging for diagnosis.** Three-dimensional CT-lymphography reconstructed from the first post-contrast images **(a)**. Lymphatic vessels drained into a single axillary sentinel node (arrow). Images of CT-lymphography **(b)** and T2*-weighted axial MR images **(c)** at the same level were compared to specify the node (arrow) on T2*-weighted axial MR imaging corresponding to the sentinel node (arrow) identified by CT-lymphography. The node (arrow) showed high-signal intensity before administration of superparamagnetic iron oxide (SPIO). **(d)** After administration of SPIO, the node showed homogenous low signal intensity and was diagnosed as benign (arrow).

3D-CT images were reconstructed from the post-contrast CT images at each time point with volume-rendering techniques and, if necessary, a workstation (GE Advantage Workstation, version 4.3; GE Healthcare, Milwaukee, WI, USA) was used to further examine lymph flow and the sentinel node (Figure 1a).

### MR imaging

MR images were obtained using a 1.5 T imaging system (Sonata/Symphony; Siemens, Erlangen, Germany) with a 12-channel matrix body coil. T1-weighted axial images were obtained from the upper thorax to axillary lesions (repetition time in msec (TR), 140; echo time in msec (TE), 1.88; slice width, 4 mm; interslice gap, 0 mm; number of acquisitions, one; field of view, 28 × 28 cm; matrix, 141 × 256); T2-weighted axial images were obtained through the axilla (TR, 4,000; TE, 85 effective time; echo train length, 11; slice width, 4 mm; interslice gap, 0 mm; number of acquisitions, one; field of view, 25 × 25 cm; matrix, 250 × 384). Additional nodal imaging sequences included T2*-weighted gradient echo images in the axial plane (TR, 613; TE, 30; flip angle, 30; slice width, 4 mm; interslice gap, 0 mm; number of acquisitions, one; field of view, 25 × 25 cm; matrix, 230 × 384).

Each patient was placed in the supine position in the adducted arm position. A 40 μl aliquot of SPIO (Resovist; FUJIFILM RI Farma Co., Ltd., Kyobashi, Tokyo), containing 1.115 mg iron, was diluted in 20 ml normal saline. After local anesthesia with subcutaneous injection of 2 ml of 2% procaine hydrochloride, 6 ml SPIO, containing 0.3345 mg iron, was injected intradermally into the skin overlying the breast tumor and into the subareolar skin. The injection sites of SPIO were gently massaged for one minute. At 18 to 24 hours after the administration of SPIO, the T1-, T2- and T2*-weighted sequences used for interpretation of the lymph node status were repeated. The MR imaging interval was determined by referring to the report of the study using USPIO [15]. T1-weighted and T2-weighted images were used for anatomic localization; the T1-weighted image in particular allows the shape and size of nodes clearly.

Images of the CT-LG (Figure 1b) and T2*-weighted axial MR images (Figure 1c) in the adducted arm position at the same level were compared to specify the node on T2*-weighted axial MR imaging corresponding to the sentinel node identified by CT-LG. If necessary, a merged image of the CT-LG and T2*-weighted axial MR images was obtained on a workstation (PEGASYS; ADAC, Milpitas, CA, USA), with the help of small plastic bullets.

**Figure 2:**
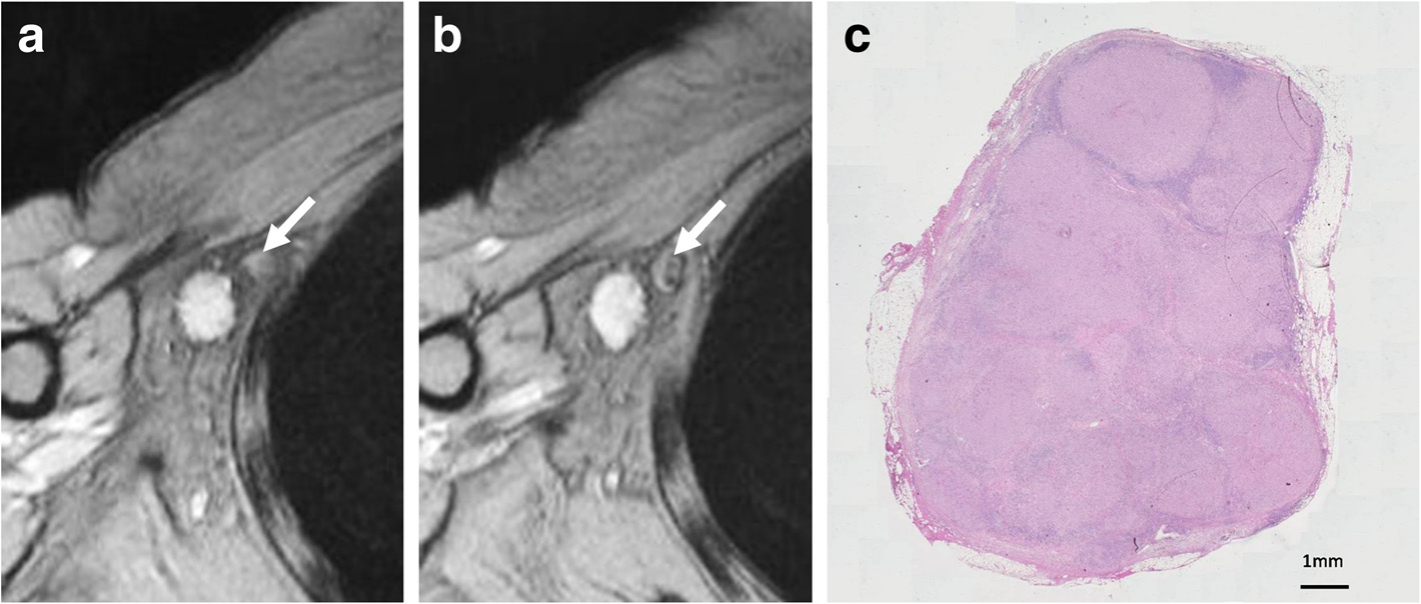
**Pattern of uniform high-signal intensity on SPIO-enhanced MR imaging. (a)** The node (arrow) showed high-signal intensity before administration of superparamagnetic iron oxide (SPIO). **(b)** After administration of SPIO, the node showed uniform high-signal intensity and was diagnosed as malignant (arrow). **(c)** Histological findings confirmed it as malignant. This node was almost entirely replaced by metastatic tissue.

**Figure 3:**
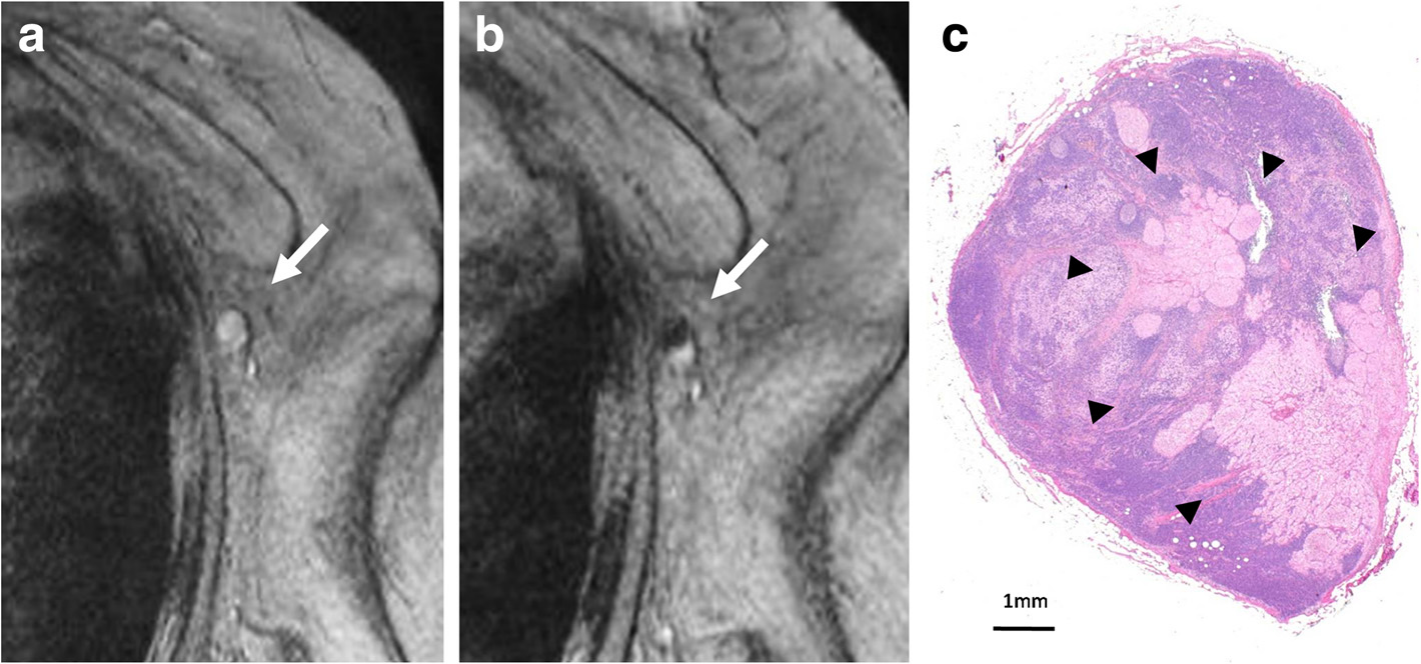
**Pattern of partial high-signal intensity involving more than 50% of the node on SPIO-enhanced MR imaging. (a)** The node showed high-signal intensity before administration of superparamagnetic iron oxide (SPIO). **(b)** After administration of SPIO, the node showed partial high-signal intensity involving more than 50% and was diagnosed as malignant (arrow). **(c)** Histological findings showed the presence of macro-metastases within the node (arrowheads).

**Figure 4:**
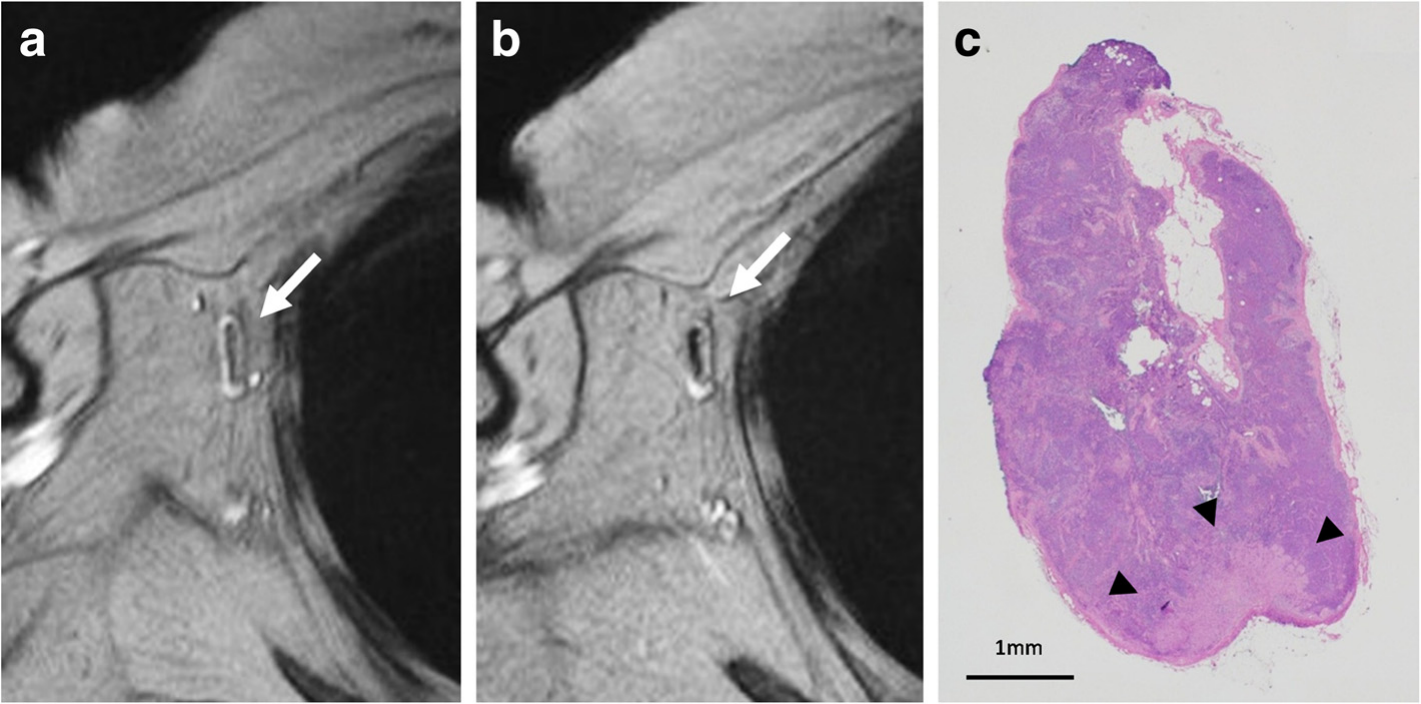
**Pattern of partial high-signal intensity involving less than 50% of the node on SPIO-enhanced MR imaging. (a)** The node showed high-signal intensity before administration of superparamagnetic iron oxide (SPIO). **(b)** After administration of SPIO, the node showed partial high-signal intensity involving less than 50% and was diagnosed as malignant (arrow). **(c)** Histological findings showed the presence of micro-metastases within the node (arrowheads).

Nodes were evaluated on pre- and post-SPIO images by 1 reader (K.M.). Visual analysis was based on the diagnostic guidelines previously reported by Anzai et al. [16]. In brief, a node was considered non-metastatic if it showed homogenous low-signal intensity (Figure 1d) and metastatic if the entire node or more than 50% of the node has high-signal intensity on post-SPIO MR imaging compared with the signal intensity on pre-SPIO images. A node was considered possibly metastatic if less than 50% of the node has high-signal intensity [16]. In this study, patterns of SPIO uptake for positive sentinel nodes were classified into three referring to the guideline [16]; uniform high-signal intensity, partial high-signal intensity involving more than 50% of the node, and partial high-signal intensity involving less than 50% of the node (Figures 2, 3 and 4).

### Surgery

Sentinel node biopsy was performed as described previously [17-19]. In brief, intradermal or intradermal and subareolar injection of 0.3 ml of 37 MBq (1 mCi) Tc-99 m tin colloid the day before surgery and peritumoral or intradermal and subareolar injection of 5 ml indocyanine green (ICG, Diagnogreen 0.5%; Daiichi Pharmaceutical Co. Ltd., Nihonbashi, Tokyo, Japan) 10 minutes before surgery were performed, and then the injection site was massaged manually for one minute. Lymphoscintigraphy was performed 2–3 hours after the radioisotope injection.

Breast surgery was performed before axillary surgery in all patients to minimize the influence of radioactivity from the injection site [17-19]. Hot nodes were identified using a gamma probe (neo2000; Neoprobe Corporation, Dublin, OH, USA). Dyed and/or hot nodes located just under the markers using CT images were defined as sentinel nodes and were removed.

### Histopathology

Sentinel nodes were serially sectioned at 2 mm intervals. Hematoxylin and eosin sections of these nodes were prepared from each 2-mm slice. When these nodes were tumor-negative in paraffin sections, an additional 4-μm section was cut and stained with immunohistochemistry (IHC) using the avidin-biotinylated peroxidase complex technique with the mouse monoclonal antibody against cytokeratin (NCL-CK19; Novocastra Laboratories Ltd., Newcastle, UK or AE1/AE3; Thermoelectron Corp., Waltham, MA, USA). On the basis of the 6th edition of the Union Internacional Contra la Cancrum TNM categories, metastatic nodes were categorized according to the degree of metastatic burden as follows: macrometastases (>2 mm) and micrometastases (0.2 to 2.0 mm). Nodes with isolated tumor cells identified by IHC were considered to be metastasis-negative [20]. The long axis of the area of high-signal intensity was compared with the pathological size of the metastases.

### Statistical analysis

Spearman’s rank-order correlation was used for statistical analysis.

## Results

The mean age of the 150 patients was 56.0 (range, 31–79) years and the mean pathologic tumor size was 19.4 (range, 0.2–60) mm. Patient and tumor characteristics are summarized in Table 1. The mean number of sentinel nodes identified by CT-LG was 1.15 (range 1–3). The mean size of sentinel nodes was 9.6 (range, 4–25) mm. Thirty-three pathologically positive sentinel nodes from 30 patients were evaluated. Four false-negative patients were excluded. Three patterns of SPIO uptake were demonstrated for positive sentinel nodes. Six nodes (18.2%) showed uniform high-signal intensity, 17 nodes (51.5%) showed partial high-signal intensity involving more than 50% of the node, and 10 nodes (30.3%) showed partial high-signal intensity involving less than 50% of the node. High-signal intensity patterns that were uniform or involved more than 50% of the node were observed in 23 nodes that contained macro-metastases and no node that contained micro-metastases, while high-signal intensity patterns involving less than 50% of the node were observed in 2 nodes that contained macro-metastases and 8 nodes that contained micro-metastases. When the area of high-signal intensity was compared with the pathological size of the metastases, a pathologic >2 mm sentinel node metastases correlated with the area of high-signal intensity, however, a pathologic ≤2 mm sentinel node metastases did not. (r_s_ = 0.482, p = 0.015; r_s_ = 0.309, p = 0.457, respectively).

**Table 1:** Patient characteristics

	**No. of patients**	**%**
Age, years		
<50	45	30.0
≥50	105	70.0
Tumor size, cm		
≤2	95	63.3
>2, ≤5	53	35.3
>5	2	1.3
Tumor location		
Upper outer	82	54.7
Upper inner	30	20.0
Lower outer	24	16.0
Lower inner	4	2.7
Central	8	5.3
Multicentric	2	1.3
Tumor histology		
Invasive ductal	132	88.0
Invasive lobular	7	4.7
Ductal carcinoma in situ	7	4.7
Others	4	2.7
Type of surgery		
Lumpectomy	146	96.7
Mastectomy	4	3.3
Estrogen receptor		
Positive	123	82.0
Negative	27	18.0
HER-2/neu		
Positive	19	12.7
Negative	131	87.3

## Discussion

The patterns of contrast enhancement have been demonstrated to distinguish between malignant and benign lymph nodes using USPIO-enhanced MR images [16]. A lymph node with an area of high-signal intensity encompassing the entire node or a portion of it was considered metastatic according to the diagnostic guidelines for USPIO-enhanced MR imaging, which are based on qualitative analysis of the results [16]. If there is no blackening of the node or if the node is hyperintense to surrounding tissue, or the node has central high-signal with darkening along the peripheral rim, or partial darkening whereby more than 50% of the node has an area of high-singal intensity, a node is diagnosed as metastatic. If less than 50% of the node has high-signal intensity, it is possibly metastatic. If the node has overall dark signal intensity, it is diagnosed as non-metastatic. A larger area of high-signal intensity within the node was reported to be more likely to be metastatic on USPIO-enhanced MR imaging. Lahaye et al. reported that an estimated area of high-signal intensity within the node that was more than 30% was highly predictive of a metastatic node, with a sensitivity of 93% and a specificity of 96% in patients with primary rectal cancer [21]. They demonstrated that the most accurate and practical predictive criterion is the estimation of the percentage of high-signal intensity within the node on USPIO-enhanced MR imaging. In the present study, we classified the patterns of SPIO uptake for positive sentinel nodes into three; uniform high-signal intensity, partial high-signal intensity involving more than 50% of the node, and partial high-signal intensity involving less than 50% of the node. We demonstrated that high-signal intensity patterns that were uniform or involved more than 50% of the node were observed in nodes with macro-metastases (Figures 2 and 3). High signal intensity patterns involving less than 50% of the node were often observed in nodes with micro-metastases (Figure 4). When the area of high-signal intensity was compared with the pathological size of the metastases, a pathologic > 2 mm sentinel node metastases correlated with the area of high-signal intensity, however, a pathologic ≤ 2 mm sentinel node metastases did not. The size of small metastatic foci could not be assessed because MR imaging had limited resolution in the present setting. It may be difficult to detect micro-metastases with a section thickness of 4 mm at this resolution on 1.5 T MR images. Interstitial administration of SPIO leads to excessive dosage or a high concentration of SPIO, which may conceal some micro-metastatic foci, resulting in underestimation of the size of metastatic foci, while the fatty hilum of a node, which coexists with metastases, may mimic a metastatic deposit, resulting in overestimation of the size of the metastatic foci. In our previous study, MR imaging with interstitial injection of SPIO was evaluated for the detection of metastases in sentinel nodes, which were localized by CT-LG in 102 patients with breast cancer [13]. The sensitivity, specificity, and accuracy of MR imaging for the diagnosis of sentinel node metastases were 84%, 91%, and 89%, respectively. In 40% of patients with micro-metastases, metastases were not detected, but all patients with macro-metastases were successfully identified. False negatives may be due to micro-metastases. Of the 7 false-positive results, 6 were due to a prominent fatty hilum. False positives may be due to prominent fatty tissue and insufficient transition of SPIO to sentinel nodes. Fat-saturated images, a 3 T MR system and special coil for MR imaging may be needed to clearly identify small metastatic foci.

However, the clinical implication of micro-metastases is debatable. De Boer et al. reported that the presence of both isolated tumor cells and micro-metastases was associated with reduced disease-free survival among patients who did not receive systemic adjuvant therapy [22]. In patients with isolated tumor cells and micro-metastases who received adjuvant therapy, disease-free survival was improved. In the systematic review, the presence of micro-metastases in axillary lymph nodes detected on single-section examination was associated with poorer disease-free and overall survival [23], while Hansen et al. reported that patients with micro-metastases do not have a worse disease-free or overall survival than sentinel node-negative patients [24]. Whether intensive identification of the existence of small disease foci is needed in clinical practice is an urgent problem.

There were some limitations to our study. There was a relatively small number of metastatic nodes in our series. This was due to the selection of patients with T1-2 breast cancers and clinically negative nodes, who do not have many metastatic axillary nodes. In addition, it is unclear whether the 50% cut-off value was appropriate. The 50% cut-off value was applied according to the diagnostic guidelines for USPIO-enhanced MR imaging, and for high-signal intensity in which more than 50% of the node was observed in sentinel nodes with macro-metastases [16]. A larger study is needed to find the most appropriate cut-off value to confirm the results of our study.

## Conclusions

High-signal intensity patterns that are uniform or involve more than 50% of the node are features of nodes with macro-metastases. The area of high-signal intensity correlated with the pathological size of metastases for nodes with metastases >2 mm in this series.

## Abbreviations

SPIO: Superparamagnetic iron oxide; USPIO: Ultrasmall superparamagnetic iron oxide; CT: Computed tomography; MR: Magnetic resonance; CT-LG: Computed tomography-lymphography; TR: Repetition time; TE: Echo time; ICG: Indocyanine green; IHC: Immunohistochemistry.

## Competing interests

The authors declare that they have no competing interests.

## Authors’ contributions

KM contributed to the conception and design of the study, data analysis and drafted the manuscript. TI and SH contributed the analysis and interpretation of the data of MR imaging. HS contributed the analysis and interpretation of the data of CT. AN provided methodological advice. TH and KN contributed to the conception and design of the study, analysis and interpretation of the data. All authors read and approved the final manuscript.

## Pre-publication history

The pre-publication history for this paper can be accessed here:

http://www.biomedcentral.com/1471-2342/13/32/prepub
